# Naturally Acquired Kelch13 Mutations in Plasmodium falciparum Strains Modulate *In Vitro* Ring-Stage Artemisinin-Based Drug Tolerance and Parasite Survival in Response to Hyperoxia

**DOI:** 10.1128/spectrum.01282-21

**Published:** 2022-09-12

**Authors:** Sandra Duffy, Vicky M. Avery

**Affiliations:** a Discovery Biology, Griffith University, Nathan, Queensland, Australia; b School of Environment and Science, Griffith University, Nathan, Queensland, Australia; Weill Cornell Medicine

**Keywords:** artemisinin tolerance, hyperoxia, K13 mutation, *Plasmodium falciparum*, genetic background

## Abstract

The ring-stage survival assay was utilized to assess the impact of physiological hyperoxic stress on dihydroartemisinin (DHA) tolerance for a panel of Plasmodium falciparum strains with and without Kelch13 mutations. Strains without naturally acquired Kelch13 mutations or the postulated genetic background associated with delayed parasite clearance time demonstrated reduced proliferation under hyperoxic conditions in the subsequent proliferation cycle. Dihydroartemisinin tolerance in three isolates with naturally acquired Kelch13 mutations but not two genetically manipulated laboratory strains was modulated by *in vitro* hyperoxic stress exposure of early-ring-stage parasites in the cycle before drug exposure. Reduced parasite tolerance to additional derivatives, including artemisinin, artesunate, and OZ277, was observed within the second proliferation cycle. OZ439 and epoxomicin completely prevented parasite survival under both hyperoxia and normoxic *in vitro* culture conditions, highlighting the unique relationship between DHA tolerance and Kelch13 mutation-associated genetic background.

**IMPORTANCE** Artemisinin-based combination therapy (ACT) for treating malaria is under intense scrutiny following treatment failures in the Greater Mekong subregion of Asia. This is further compounded by the potential for extensive loss of life if treatment failures extend to the African continent. Although Plasmodium falciparum has become resistant to all antimalarial drugs, artemisinin “resistance” does not present in the same way as resistance to other antimalarial drugs. Instead, a partial resistance or tolerance is demonstrated, associated with the parasite’s genetic profile and linked to a molecular marker referred to as K13. It is suggested that parasites may have adapted to drug treatment, as well as the presence of underlying population health issues such as hemoglobinopathies, and/or environmental pressures, resulting in parasite tolerance to ACT. Understanding parasite evolution and control of artemisinin tolerance will provide innovative approaches to mitigate the development of artemisinin tolerance and thereby artemisinin-based drug treatment failure and loss of life globally to malaria infections.

## INTRODUCTION

Artemisinin (ART) based combination therapy (ACT) for the control and cure of Plasmodium falciparum (*Pf*) malaria has dramatically reduced both incidence and mortality rates globally (1). However, there is great concern regarding the emergence of ACT treatment failure due to underlying ART tolerance or partial resistance within the Greater Mekong Subregion of Asia (GMS). This is referred to as an extended parasite clearance time (PCT) or lower clearance rate after artesunate (ATS) monotherapy (2, 3). Due to the observed extended PCT with ATS monotherapy, ACT, consisting of an ART derivative and a longer-lasting partner drug, became the WHO-recommended treatment for noncomplicated malaria. However, the underlying ART partial resistance, or reduced sensitivity, is selecting for partner drug resistance and resulting in ACT treatment failures in the GMS (4, 5). Single nucleotide polymorphisms in a Kelch13 propeller domain (PF3D7_1343700), commonly referred to as K13, were identified as a molecular marker for partial ART resistance and associated with extended parasite clearance times by Ariey et al. (6). Parasites from patients with extended PCT do not demonstrate a significant shift in *in vitro* 50% inhibitory concentrations (IC_50_) compared to those cleared quickly. The *in vitro* ring-stage survival assay (RSA), which detects early-ring-stage parasite survival after 6 h exposure to a clinically relevant 700 nM dose of dihydroartemisinin (DHA) (7, 8), is now an accepted component of decreased ART sensitivity and K13-associated extended-PCT surveillance. Initially, K13-delayed PCT was observed in western Cambodia (2, 3), but it has subsequently evolved independently throughout the GMS (9, 10) and more recently in Amazonia (11), Papua New Guinea (12), and Rwanda (13–15).

The K13 protein is involved with endocytosis (16, 17), with K13 mutations resulting in reduced protein abundance (18–21), leading to enhanced parasite survival in the RSA. It is postulated that this is due to the reduction in hemoglobin catabolism necessary for the activation of ART (21, 22) and subsequent generation of reactive oxygen species (ROS) (22), thereby limiting the alkylation of a wide range of proteins (23). Currently, K13 mutant parasites are still predominantly contained within the GMS and are not prevalent on the African continent. Understanding how and why these parasites have independently developed globally is necessary to prevent further adaptation of ART-tolerant *Pf* parasites into multidrug-resistant forms. To address this, extensive molecular studies using clinical isolates, such as the studies by Zhu et al. (24, 25), were undertaken to expand the knowledge base within this area (25). However, focused *in vitro* studies may provide further clues to the mechanisms underlying ART tolerance, often hidden within such extensive data sets (18, 26, 27).

A defined genetic background is associated with predisposing *Pf* to ART tolerance (28, 29). This genetic background has developed at a population level within distinct geographical locations (30, 31) and is the backbone upon which K13 mutation-associated ART partial resistance and ACT treatment failures have arisen. Genome-wide association studies identified several markers associated with pre-K13 acquisition (32), including but not limited to fd (ferredoxin), arps10 (apicoplast ribosomal protein S10), mdr2 (multidrug resistance protein 2), and crt (chloroquine resistance transporter). The population dynamics of these parasites continue to evolve, under various selective pressures, encompassing resistance to other antimalarial drugs, including piperaquine (8, 33), resulting in the multidrug-resistant parasite lineage KEL1/PLA1 (34), now fixed within the GMS parasite population.

An underlying outcome of functional ART tolerance studies is the implication of many parasite survival mechanisms, including enhanced anti-oxidative stress and DNA repair, aiding parasite recovery after DHA exposure. This includes *in vitro* laboratory-generated resistant parasites without acquired K13 mutations and those in founder populations prone to K13 mutations (35–39). *In vitro* evolution of parasite resistance to ART derivatives, due to drug exposure, has identified numerous targets, inclusive of mdr1, pfcrt (40), coronin (41), and atg18 (42). However, only one study identified a mutation in the K13 propeller gene (F32-ART M476I) (6) resulting from *in vitro* drug resistance evolution studies.

*In vitro* culturing conditions employed for the generation of F32-ART K13 M476I were reported as 37°C in an atmosphere of 5% CO_2_ (43), i.e., with oxygen levels reflecting those of atmospheric air (approximately 21% O_2_). P. falciparum, a microaerophilic organism (44), is most often cultured in O_2_ levels between 1 and 5% (6, 7, 18, 44–47), which is in alignment with physiological O_2_ levels of the human host bloodstream (48, 49). Normoxia, defined as normal oxygen level, is often used interchangeably to describe atmospheric air and that present within CO_2_ tissue culture incubators. However, *in vivo* asexual *Pf* parasites reside within red blood cells (RBC), which have a physiological normoxia of approximately 4 to 7.5% O_2_ (48); thus, normoxia in the context of the current study is defined as 5% O_2_. Atmospheric air (21% O_2_) is referred to here as hyperoxia, i.e., elevated oxygen levels in comparison to normoxia. Hyperoxia employed for *Pf in vitro* culture is reported to induce oxidative stress within *Pf* (50), demonstrating a coordinated and regulated response by selectively regulating components of the antioxidant defense machinery and heat shock chaperones (51). This being the case, continuous *in vitro* culturing at levels of O_2_ higher than physiological normoxia for the generation of F32-ART K13 M476I (43) may be considered a serendipitous model system demonstrating K13 mutation acquisition on a backbone of adaptation to oxidative stress, with the K13 M476I mutation acquired after approximately 30 cycles of drug treatment (<1 year’s selection). Several previous studies investigated the influence of oxygen concentration on both parasite proliferation characteristics (50–53) and compound activity (52, 54), for both laboratory strains (50–53) and clinical isolates (52). However, although the relationship between elevated antioxidant stress capability and ART tolerance has been demonstrated *in vitro*, phenotypic *in vitro* evidence has not previously been studied to any great extent. This is particularly the case with regard to incorporating levels of O_2_ considered physiologically hyperoxic, K13 mutations and the associated genetic background, and the collective impact on DHA tolerance in the early ring stage of parasite development.

To investigate the relationship between *Pf in vitro* physiological hyperoxia, K13 mutations, a proposed genetic background associated with the acquisition of K13 mutations, and DHA tolerance of very young ring-stage *Pf* parasites, a panel of 12 *Pf* strains with and without K13, fd, crt, mdr2, arps10, and nif4 mutations, plus laboratory strains (Table 1), was employed. These strains were selected based on their prior use to define the relationship between K13 mutations with delayed PCT (Cam2 C580Y [MRA-1236], Cam3.1 R593T [MRA-1240], Cam5 I543T [MRA-1241], IPC3663 [MRA-1237], and CamWT [MRA-1250]) (6) and validation of ART tolerance of early-ring-stage parasites (all strains apart from 3D7 [MRA-102]) (55). Cam2 C580Y, Cam3.1 R539T, CAM5 I543T, CamWT^C580Y^ (MRA-1251), and Dd2^R539T^ (MRA-1255) all demonstrate ART tolerance in the RSA, whereas IPC3663, CamWT, Dd2, and 3D7 are ART sensitive (6, 55). The ART-tolerant strains have also been utilized in compound screening (56–58), identifying the association between a histone acetyltransferase (PfGCN5) and the regulation of stress-responsive genes (39), as well as the relationship between the RNA polymerase (Pol) III repressor Maf1 and isoleucine starvation and ART resistance (59). The 12 strains are therefore highly characterized and validated for their application in ART tolerance studies. Of note, all strains are available through BEI Resources, thereby providing a point of reference for retrospective and future studies on ART tolerance.

**TABLE 1:** Parasite strain nomenclature and associated genetic profiles

Group	Strain	BEI catalog no.	Type^*a*^	K13 mutation	% survival in RSA		Presence of mutation (other than K13)^*b*^				
					Original	BEI batch data	mdr2 T484I	nif4 V1157L	fd D193Y	pfcrt I356T	arps10 V127M
A	Cam2 C580Y	MRA-1236	Isolate	C580Y	27.3	16.3	Y	Y	Y	Y	Y
	Cam2^rev^	MRA-1254	Isolate, GMO	WT	2.4	4.65	Y	Y	Y	Y	Y
	Cam3.1 R539T	MRA-1240	Isolate	R539T	88.2	69.4	Y	Y	Y	Y	Y
	Cam3.1^rev^	MRA-1252	Isolate, GMO	WT	0.3	2.68	Y	Y	Y	Y	Y
	Cam5 I543T	MRA-1241	Isolate	I543T	49.3	31.5	Y	Y	Y	Y	Y
	Cam5^rev^	MRA-1253	Isolate, GMO	WT	0.3	0.29	Y	Y	Y	Y	Y
B	Cam WT	MRA-1250	Isolate	WT	0.6	0.65	X	X	X	X	X
	Cam WT^C580Y^	MRA-1251	Isolate, GMO	C580Y	8.9	17.2	X	X	X	X	X
	Dd2	MRA-156	Lab strain	WT	ND	ND	X	X	X	X	X
	Dd2^R539T^	MRA-1255	Lab strain, GMO	R539T	19.4	9.4	X	X	X	X	X
	IPC3663	MRA-1237	Isolate	WT	0.1	0.6	X	X	X	X	X
	3D7	MRA-102	Lab strain	WT	ND	ND	X	X	X	X	X

a GMO, genetically modified organism.

b Y indicates genetic mutation present (12). X indicates no association with delayed parasite clearance time and no documented associated genetic background. The mutations listed are only a small sample of the genetic backgrounds associated with K13 mutations either before or after K13 acquisition.

This study aimed to perform a preliminary investigation into the influence of physiological hyperoxia, as a stimulus of oxidative stress (50, 60), on DHA tolerance in relation to K13 mutations and a proposed naturally acquired K13-associated genetic background. Based on the parent strain K13 genetic background (naturally acquired K13 mutation or K13 wild type [WT]), the 12 strains were separated into two groups of 6 strains. Group A consisted of strains with a K13 mutation genetic background, including Cam2 C580Y, Cam3.1 R539T, Cam5 I543T, and strains with the respective reversal of the K13 mutations to WT (Cam2 WT^rev^ [MRA-1254], Cam3.1 WT^rev^ [MRA-1252], and Cam5 WT^rev^ [MRA-1253]) (55). Group B consisted of K13 WT genetic background, IPC3663, Cam WT, 3D7, and Dd2 laboratory strains and 2 genetically modified strains, CamWT^C580Y^ (MRA-1251 isolate) and Dd2^R539T^ (MRA-1255, laboratory strain), which have the K13 C580Y or K13 R539T mutation inserted in the Cam WT or Dd2 parental strains, respectively (55).

As previous studies have demonstrated altered *Pf* proliferation characteristics when transitioning from low to higher O_2_ concentrations (50, 53, 54) the impact of hyperoxia on parasite proliferation was first investigated for the 12 strains, before investigation of the impact on DHA tolerance in the RSA.

## RESULTS

### Effects of first- and second-cycle hyperoxic exposure on parasite proliferation.

**FIG 1:**
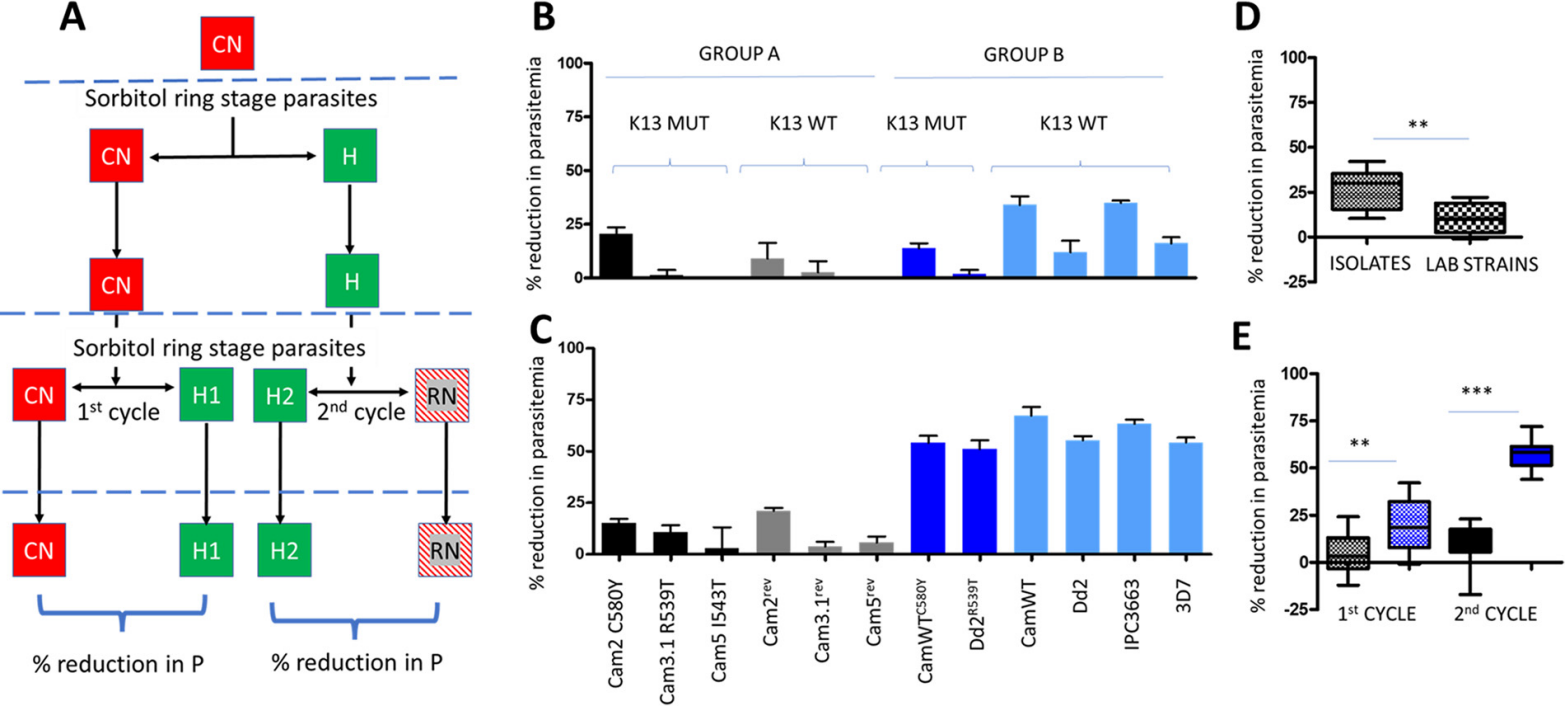
Comparison of first versus second cycle *in vitro* parasite proliferation reduction in response to hyperoxia, expressed as percent reduction in parasitemia in comparison to normoxia controls. (A) Flow chart of hyperoxia exposure of cultures. Ring-stage parasites were obtained from the constant normoxia control (CN) at schizont rupture (dashed blue line). Half the culture volume was exposed to hyperoxia (H; green squares), and the remainder was maintained in normoxia (CN) for completion of the parasite development cycle. After the next schizont rupture, ring-stage parasites were isolated and once again divided in half, with half being placed under hyperoxic conditions and half under normoxic conditions, resulting in a total of 4 cultures. CN and H1 represent a first hyperoxia cycle comparison culture set, and H2 and RN (return to normoxia) the second hyperoxia exposure cycle comparison set. (B) First-cycle reduction in parasitemia for group A (black and gray) versus group B (dark and light blue columns) *Pf* strains. Blue brackets define groups A and B and the K13 status of the 12 strains. (C) Second-cycle reduction in parasitemia for group A and group B *Pf* strains. (D) Comparison of group B first-cycle reduction in parasitemia between clinical isolates versus laboratory strains. (E) Statistical significance between group A and group B reduction in parasitemia within the first or second cycle of proliferation. Statistical significance between group A and group B (panels D and E) was determined using the two-tailed nonparametric Mann-Whitney *U* test with 95% confidence intervals. **, *P* = 0.005; ***, *P* = 0.0005.

Twelve *Pf* strains with and without K13 mutations (Table 1) were evaluated for reduction in parasitemia under conditions of normoxia (5% O_2_, 5% CO_2_; control conditions) versus hyperoxia (21% O_2_, 5% CO_2_) for either 1 or 2 cycles of proliferation (Fig. 1). A single cycle of proliferation was approximately 48 h, although some variation between strains was observed.

Significant differences in reduction of parasitemia after the first (Mann-Whitney *U P* = 0.0034) and second (Mann-Whitney *U P* = 0.0001) proliferation cycles in hyperoxia were observed between groups A and B (Fig. 1B, C, and E). While group A strains maintained similarly low levels of reduction in parasitemia between the first and second cycles of hyperoxia (Fig. 1B and C), group B demonstrated greater reduction in parasitemia after the second cycle (Fig. 1C) than after the first cycle. Group B (K13 WT genetic background) consisted of two subgroups (Fig. 1D) based on the levels of reduction in parasitemia observed (Mann-Whitney *U P* = 0.004) after the first cycle of hyperoxia; the Cambodian clinical isolates (Cam WT, Cam WT^C580Y^, and IPC3663) and laboratory strains (Dd2, Dd2^R539T^, and 3D7). In this study, the K13 WT Cambodian clinical isolates Cam WT and IPC3663 were more susceptible to hyperoxia than the standard laboratory strains, Dd2 and 3D7. Although the insertion of K13 mutations into Cam WT and Dd2 (Cam WT^C580Y^ and Dd2^R539T^, respectively) resulted in both parasites having a lesser reduction in parasitemia than their parental strains, only the Cam WT^C580Y^ response was significant (Student’s *t* test, 95% confidence intervals, *P* = 0.0128). A K13-associated genetic background (group A) played a significant role in strain-specific enhanced capability to transition from low- to high-oxygen-generated stress after both the first (Mann-Whitney *U P* = 0.0034) and second (Mann-Whitney *U P* = 0.0001) cycles of proliferation (Fig. 1E), compared to K13 WT strains (group B).

A key outcome from this investigation was the determination that K13 mutations do not confer the ability to maintain parasitemia when acutely transitioning from low- to higher-O_2_-stress environments. Two observations point to this conclusion. First, all six strains in group A, consisting of three sets with K13 mutations and their reversal to WT isogenic strains, showed no difference (Mann-Whitney *U P* = 0.7962) in their ability to transition from low- to high-O_2_ stress in an acute manner. Second, two genetically engineered K13 mutant strains from group B (Cam WT^C580Y^ and Dd2^R539T^) aligned with group B and not group A in their responses to hyperoxic exposure. Therefore, although K13 mutations confer DHA tolerance in the *in vitro* RSA, they do not, in the context of this study, provide a significantly enhanced capability for dealing with transitions from low- to high-O_2_-stress environments. This capability is likely provided by a currently undefined “K13-associated genetic background,” as indicated by the highly significant (Mann-Whitney *U P* = 0.0001) difference in reduction of parasitemia between the two groups of strains (Fig. 1E) after the second cycle of proliferation.

### Dihydroartemisinin tolerance.

All preliminary investigations regarding DHA tolerance were performed using a single strain (Cam5 I543T) with intermediate survival rate (approximately 30 to 50%) reported independently by Ariey et al. (6) and BEI Resources. Once provisional data were obtained, other strains with validated K13 mutations associated with ART tolerance were evaluated.

### (i) First- or second-cycle hyperoxia effects on *Pf* DHA tolerance.

**FIG 2:**
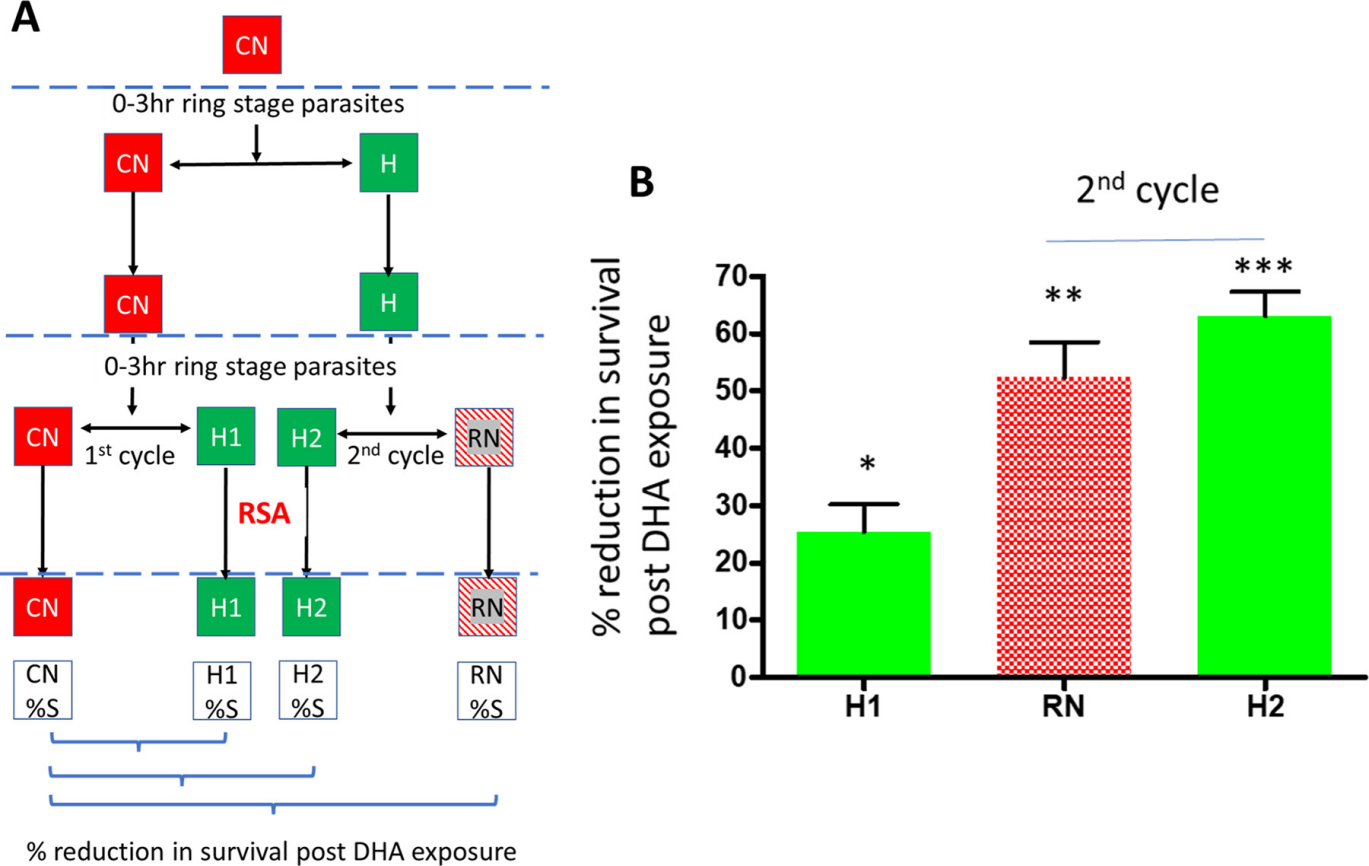
Effect of 1-cycle versus 2-cycle *in vitro* hyperoxia exposure on DHA tolerance of the K13 mutant parasite strain Cam5 I543T. (A) Experimental flow diagram. At the schizont stage (dashed blue lines), 0- to 3-h ring-stage CN parasites were isolated, and half were exposed to hyperoxia (H; green squares) while the other half remained in normoxia (CN; red squares) for the remainder of the parasite development cycle. At the following schizont stage, 0- to 3-h ring-stage parasites were isolated, and each culture was divided into two volumes for testing in the RSA, in the presence (H and H2) or absence (CN and RN) of hyperoxia. The percent reduction in survival after DHA exposure, in comparison to the continuous normoxic control (CN), was calculated from the %S values determined for each culture in the RSA. (B) Effect of hyperoxia on DHA tolerance. The first-cycle hyperoxia RSA data (H1) represents an acute exposure to both hyperoxia and DHA simultaneously with no prior hyperoxia exposure. The second-cycle data represent the effect of hyperoxia during the cycle prior to performing the RSA in normoxia (RN) or in conjunction with hyperoxia (H2). Statistical significance was calculated (Student’s *t* test, two tailed, 95% confidence intervals) was calculated for the comparison between test conditions and constant normoxia (CN). *, *P* < 0.05; **, *P* < 0.005; ***, *P* < 0.0005. Values are means (*n* = 3) and standard deviations (SD).

In response to the effect of hyperoxia on parasitemia levels (Fig. 1B, C, and E), the effect of first- and second-cycle proliferation, under conditions of hyperoxia, on Cam5 I543T DHA tolerance was assessed using the RSA (Fig. 2A). To perform this evaluation, highly synchronous 0- to 3-h ring-stage parasites were isolated by magnetic column and sorbitol lysis. The cultures were then processed as shown in Fig. 2A, and the RSA was performed with 0- to 3-h ring-stage parasites. The percentage survival for each culture condition RSA was determined and subsequently expressed as percent reduction in parasite tolerance to DHA in comparison to the continuous normoxic control (CN).

Figure 2B shows that acute hyperoxia (H1) at the time of DHA exposure had a small but significant (Student’s *t* test *P* = 0.0162) effect on parasite survival after DHA exposure (Fig. 2A [H1]). However, parasites primed for one cycle under hyperoxic conditions (Fig. 2A [H]) prior to DHA treatment demonstrated a significantly greater reduction in rates of survival of DHA either with (Fig. 2A and B [H2]) (Student’s *t* test *P* = 0.0006) or without (Fig. 2A and B [RN]) (Student’s *t* test *P* = 0.002) combined hyperoxic exposure in the second cycle in comparison to the constant normoxia control (CN).

### (ii) Influence of hyperoxia exposure time and parasite stage of development on subsequent DHA tolerance.

Once inside RBC, P. falciparum develop through ring, trophozoite, and finally schizont forms, where multiple daughter merozoites are subsequently released into the bloodstream to reinvade new RBC. The previous data (Fig. 2B) demonstrated that hyperoxic exposure during a single proliferation cycle initiated at the early ring stage (0 to 3 h) sensitized the *Pf* parasite to DHA in the next cycle of parasite development. This evaluation was therefore to determine if hyperoxic exposure initiated with 0- to 3-h ring-stage parasites was also exposure duration and parasite stage dependent.

**FIG 3:**
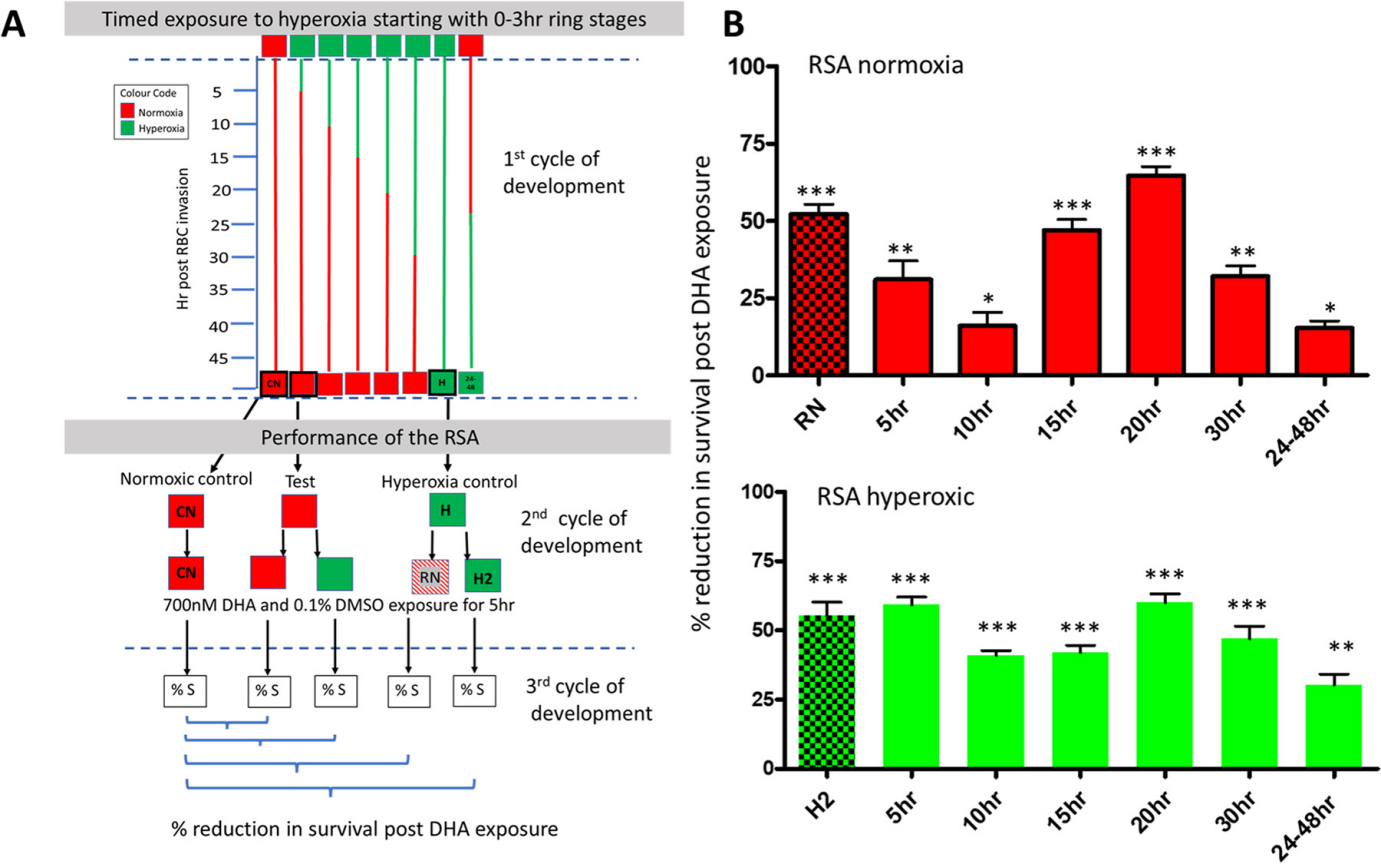
Influence of hyperoxia exposure time and parasite stage of development on subsequent DHA tolerance. (A) Experimental flow diagram. CN 0- to 3-h ring-stage parasites were divided into eight 150-mm petri dishes. At time zero (after 0- to 3-h ring-stage parasite isolation), six dishes were exposed to hyperoxia (green) and two remained in normoxia (CN and red 24 to 48 h trophozoite evaluation). After 5 h of hyperoxic exposure, a single dish was returned to normoxia, as was one dish at 5-h intervals thereafter (shown by the changing line color from red [normoxia] to green [hyperoxia]), resulting in 5-, 10-, 15-, 20-, and 30-h hyperoxia-exposed cultures in the first parasite development cycle. A single petri dish of culture was maintained under normoxic conditions until 24 h after RBC invasion and then exposed to hyperoxia for the remainder of the first cycle of parasite development. The CN control remained under normoxic conditions for the complete first development cycle to mature schizonts, and a single dish remained in hyperoxia for the complete cycle of development (H). At the schizont stage of the first cycle (dashed blue line), 0- to 3-h parasites were isolated from all 8 individual cultures. Each of the 8 cultures in the second parasite developmental cycle was evaluated in the RSA under both hyperoxic and normoxic conditions, apart from the CN control, which was tested only under normoxic conditions. The percent survival in the RSA was then used to determine the reduction in parasite survival due to the hyperoxia exposure time/condition as a percentage of the constant normoxia control: 100 − (%S test culture condition/%S CN × 100) = % reduction in survival after DHA exposure. DMSO, dimethyl sulfoxide. (B) Percentage reduction in survival after DHA exposure in comparison to the continuous normoxic culture (CN) plotted against time after RBC invasion. *n* = 3 for control sets (3 under constant hyperoxia and 3 under constant normoxia), and *n* = 3 individual biological replicates for timed cultures for each time point. Statistical significance for the reduction in parasite survival for each hyperoxia exposure duration in the first cycle of development, in either normoxia or hyperoxia in the second cycle of parasite development, was calculated in comparison to the survival of the CN control (Student’s *t* test, two tailed, 95% confidence intervals). *, *P* < 0.05; **, *P* < 0.005; ***, *P* < 0.0005. Values are means (*n* = 3) and SD. H2 and RN indicate the hyperoxia controls maintained under hyperoxia for a full cycle within the first cycle of parasite development, prior to performing the RSA in normoxia (RN) or hyperoxia (H2), i.e., equivalent to second-cycle data in Fig. 2B. Green and red “24-48hr” columns reflect the effect of hyperoxia on trophozoite stages and the subsequent influence on DHA tolerance.

The experimental design for this evaluation is presented in Fig. 3A. Two internal controls were performed in parallel: (i) constant normoxic (CN) exposure and (ii) one full cycle of hyperoxia, designated H2 and RN when tested within the subsequent RSA evaluation in hyperoxia and normoxia, respectively (Fig. 3B).

The reduction in parasite survival at each time point and condition, expressed as the percent decrease in survival in comparison to the constant normoxic control (CN), was plotted against time after RBC invasion. Reduction in parasite survival following DHA challenge, without coadministered hyperoxia during the RSA (Fig. 3B, top, red columns), was maximal by 20 h before exposure to normoxia. Extended hyperoxic exposure beyond 20 h, i.e., 30 h, resulted in a reacquisition/retention of DHA tolerance when the RSA was performed under normoxic conditions. However, continuous hyperoxic exposure for a complete cycle of parasite development into the subsequent cycle, which encompasses schizont development, with the RSA subsequently performed in normoxia (Fig. 3B [RN]), resulted in reduction in parasite survival comparable to that observed after 20 h hyperoxia exposure. Exposure of trophozoites to hyperoxia through development to schizonts in the pre-RSA cycle for 24 h resulted in minimal DHA tolerance reduction, confirming that the observed effect is specific to both ring stage and time after RBC invasion, rather than a general result of hyperoxic exposure time. However, the H2 culture (Fig. 3B, bottom), which was maintained in hyperoxia for a full development cycle, including schizonts, may indicate that hyperoxic exposure of early ring-stage parasites followed by mature schizonts may influence next-generation ring-stage DHA sensitivity. As little as 5 h hyperoxia exposure of young ring-stage parasites after RBC invasion, the cycle prior to performing the RSA in conjunction with hyperoxia, gave results comparable to those for parasites maintained continuously under hyperoxic conditions (Fig. 3B, bottom, green columns), suggesting an additive effect between hyperoxia and DHA exposure of young ring-stage parasites, irrespective of length of exposure time.

These combined data clearly indicated that hyperoxia exposure of ring-stage parasites at 0 to 20 h after RBC invasion initiated a biological response reducing future DHA tolerance (or alternatively increasing parasite sensitivity to DHA) without coadministered hyperoxic stress, possibly by an alternative mechanism to that elicited during DHA coadministration with hyperoxia.

### (iii) Effect of hyperoxia exposure to 0- to 20-h ring-stage parasites on parasite proliferation.

**FIG 4:**
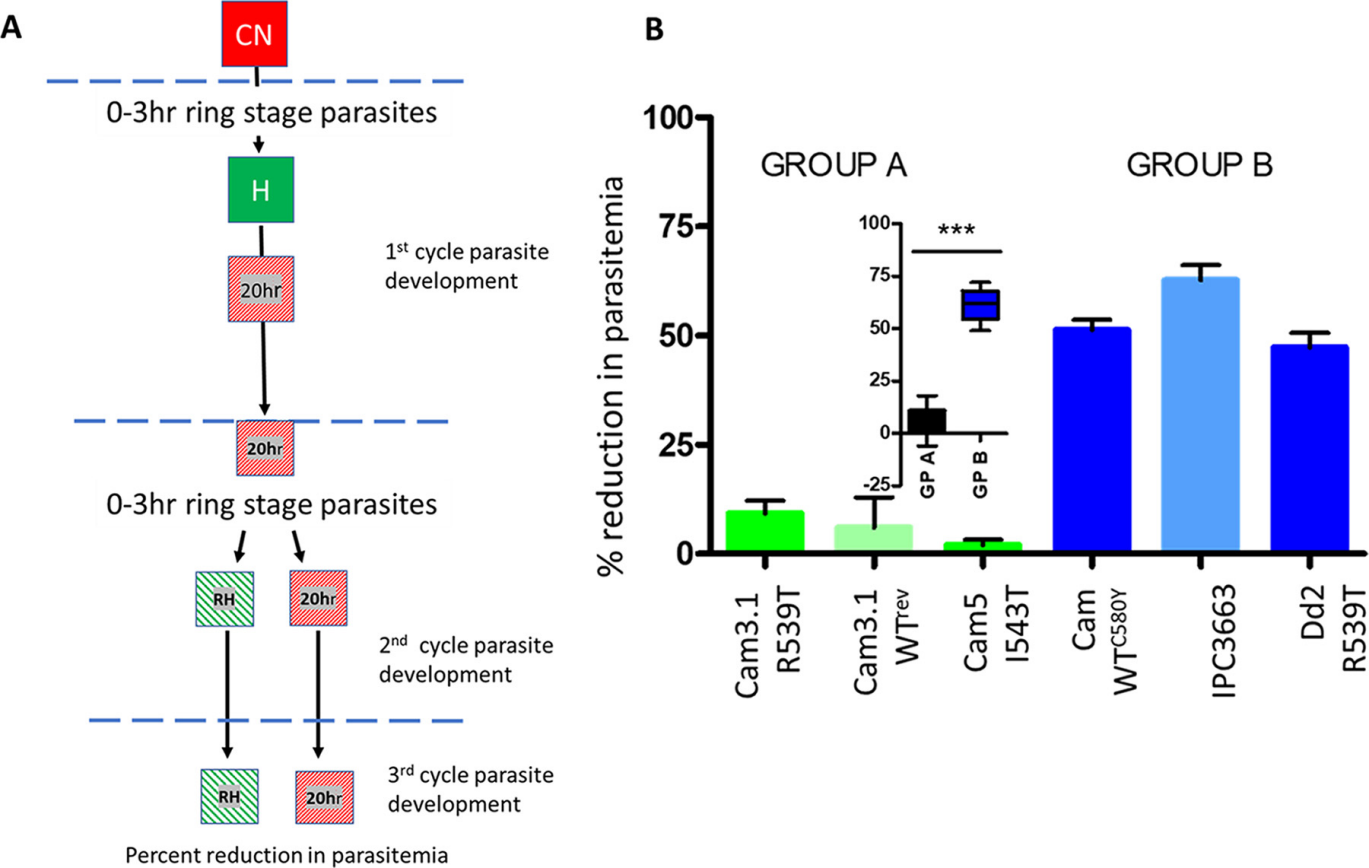
Effect of hyperoxia exposure of 0- to 20-h ring-stage parasites on parasite proliferation. (A) Experimental flow diagram. Early-ring-stage 0- to 3-h normoxia control (CN) parasite cultures were isolated and exposed to hyperoxia for the first 20 h of parasite development. The CN in this evaluation was not performed. Early-ring-stage parasites (0 to 3 h) of the 20-h-hyperoxia-exposed parasite cultures were isolated, and each culture was divided in half. One half was incubated in hyperoxia (return to hyperoxia [RH]) and one half in normoxia (20 h) for a further 65 h. (B) Influence of 0- to 20-h hyperoxic exposure on parasite proliferation for three group A strains (Cam3.1 R539T, Cam3.1WT^rev^, and Cam5 I543T) and three group B strains (Cam WT^C580Y^, IPC3663, and Dd2^R539T^), in conjunction with hyperoxia or normoxia during the second cycle of proliferation, expressed as percent reduction in parasitemia. Statistical significance (Mann-Whitney *U P* < 0.001) between groups A and B is shown in the inset (Mann-Whitney *U* test, two tailed, 95% confidence intervals). ***, *P* = 0.0005.

Having identified a link between ring-stage hyperoxia (20 h) and reduced DHA tolerance (Fig. 3B), the effect of hyperoxia ring stage-specific exposure (20 h) on parasite proliferation was assessed (Fig. 4A and B). Hyperoxic exposure of the three group A strains (Cam5 I543T, Cam3.1 R539T, and Cam3.1^rev^) for the first 20 h of parasite development did not affect parasite proliferation after a second cycle of hyperoxia, as expected. However, the group B strains IPC3663, Dd2^R539T^, and Cam WT^C580Y^ demonstrated a reduced proliferation in response to a second cycle of hyperoxia (Fig. 4A, return to hyperoxia [RH]). These data indicated that the first 20 h of parasite development under hyperoxia stress conditions played a specific role in the reduced capability of group B strains to withstand stress conditions in the subsequent proliferation cycle, resulting in a loss of parasitemia. In contrast, a subset of 3 group A strains (CAM5 I543T, Cam3.1 R539T, and Cam3.1 WT^rev^) was able to tolerate the effects of hyperoxia during the first 20 h of development, i.e., the ring stage, with no detrimental effects on proliferation after the second cycle of hyperoxia.

An intermediate conclusion drawn from these data was that both the ability to adapt to hyperoxic conditions and maintain parasitemia levels (Fig. 1B to E and 4B) and decreased DHA tolerance in response to hyperoxia (Fig. 2B and 3B) were influenced by hyperoxia exposure within the first 20 h of parasite development after RBC invasion one cycle prior to drug exposure. However, this is not governed by K13 mutations.

### (iv) Effect of 20 h hyperoxia on DHA tolerance of K13 mutation-containing parasite strains with and without associated delayed-PCT genetic background.

**FIG 5:**
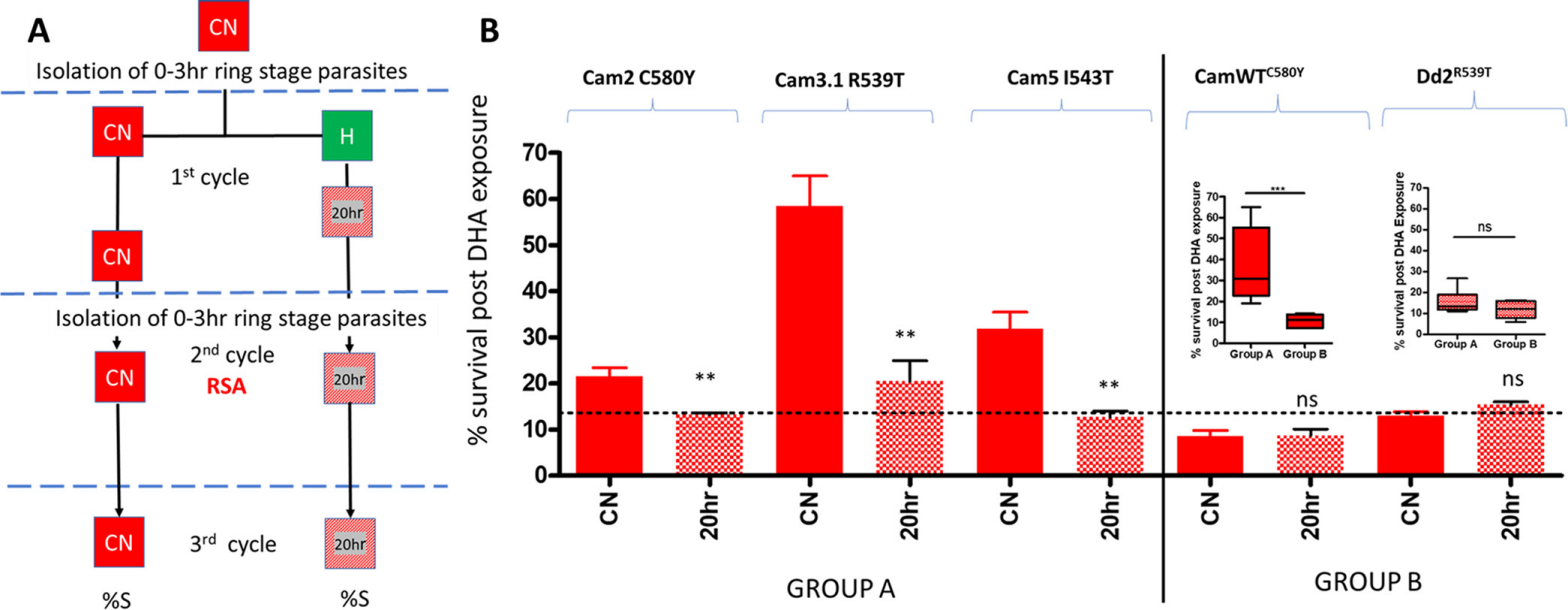
Effect of 20 h hyperoxia on DHA tolerance of K13 mutant containing parasite strains with and without associated delayed-PCT genetic background. (A) Experimental flow diagram. Control normoxia (CN) early ring-stage parasites (0 to 3 h) were isolated and exposed to hyperoxia (H) for the first 20 h of ring-stage parasite development before returning to normoxic conditions for the remainder of the first development cycle (20 h; red cross-hatched squares). The normoxic control (CN) was processed only in normoxia throughout the evaluation. At the schizont stage for the first development cycle (dashed blue line), 0- to 3-h early ring-stage parasites were isolated for both the 20-h hyperoxic (20 h)-exposed and constant normoxia control (CN) cultures. The ring-stage parasites were then tested for their DHA tolerance (RSA) in the second development cycle, all under normoxic conditions (CN and 20 h). (B) Percent survival after DHA exposure for all strains and conditions evaluated. The dashed line represents the average survival for all five strains after 20 h hyperoxia exposure in the first cycle of proliferation (average, 14.02 ± 5.1). The line, therefore, indicates a residual level of parasite survival proposed to be directly influenced by K13 mutations and not impacted by hyperoxia. *P* values were calculated using unpaired Student’s *t* test, one tailed (one directional), with 95% confidence limits using GraphPad Prism. **, *P* < 0.005; ns, not significant. Values are means (*n* = 3) and SD. (Inset) Comparison between survival control data for group A and group B (solid red) and 20-h-hyperoxic-exposure data (red hatched). Statistical significance between group A and B was calculated using the Mann-Whitney *U* two-tailed test, with 95% confidence intervals. ***, *P* = 0.0005.

The effect of 20 h hyperoxia in comparison to constant normoxia (CN) on DHA tolerance of naturally acquired K13 mutations (group A: Cam2 C580Y, Cam3.1 R539T, and Cam5 I543T) in comparison to K13 mutations genetically inserted into K13 WT genetic backgrounds (group B: Cam WT^C580Y^ and Dd2^R539T^) was evaluated. Early-ring-stage parasites (0 to 3 h) were exposed to hyperoxia for 20 h in the parasite development cycle before the RSA was performed (0- to 3-h rings) in normoxia. Data are presented (Fig. 5B) as percent survival after DHA exposure for each parasite strain and culturing condition.

The group A strains, Cam2 C580Y, Cam3.1 R539T, and Cam5 I543T, demonstrated a reduction in parasite survival after DHA treatment post after exposure to hyperoxia in the previous development cycle between 0 and 20 h after RBC invasion. In comparison, CamWT^C580Y^ and Dd2^R539T^ (group B) did not, resulting in survival rates comparable to those obtained for Cam3.1 R539T, Cam5 I543T, and Cam2 C580Y after hyperoxic exposure. Statistical analysis (Fig. 5, inset) of median normoxic control data (CN) indicated a highly significant difference between group A and group B parasites (Mann-Whitney *U P* = 0.0004). Group A normoxia control (CN) data demonstrated greater survival after DHA exposure than group B, but no significant difference between groups A and B after 20 h hyperoxic exposure (Fig. 5, inset) was observed. In Fig. 5, activity above the dotted line for normoxia controls (average survival in response to hyperoxia, 14.02% ± 5.1%) for group A strains is suggestive of genetic background modulation of DHA tolerance elicited by K13 mutations, when no prior stress was applied to the parasite prior to DHA exposure.

It is possible to conclude from these data that group A genetic background enhances parasite tolerance to hyperoxia stress and works in conjunction with the K13 mutation to enhance parasite survival to the singular stress of DHA challenge. However, when parasites were exposed to other stressors, such as hyperoxia, a cycle prior (0 to 20h post-RBC invasion) to DHA exposure, this enhanced survival was negated to a common residual parasite survival level (Fig. 5, dotted line) (average survival, 14.02% ± 5.1%), comparable to that of group B strains, irrespective of the presence of K13 C580Y, R539T, or I543T mutations.

### (v) Evaluation of hyperoxic exposure on parasite tolerance to ART, ATS, OZ277, OZ439, and the proteasome inhibitor epoxomicin.

**FIG 6:**
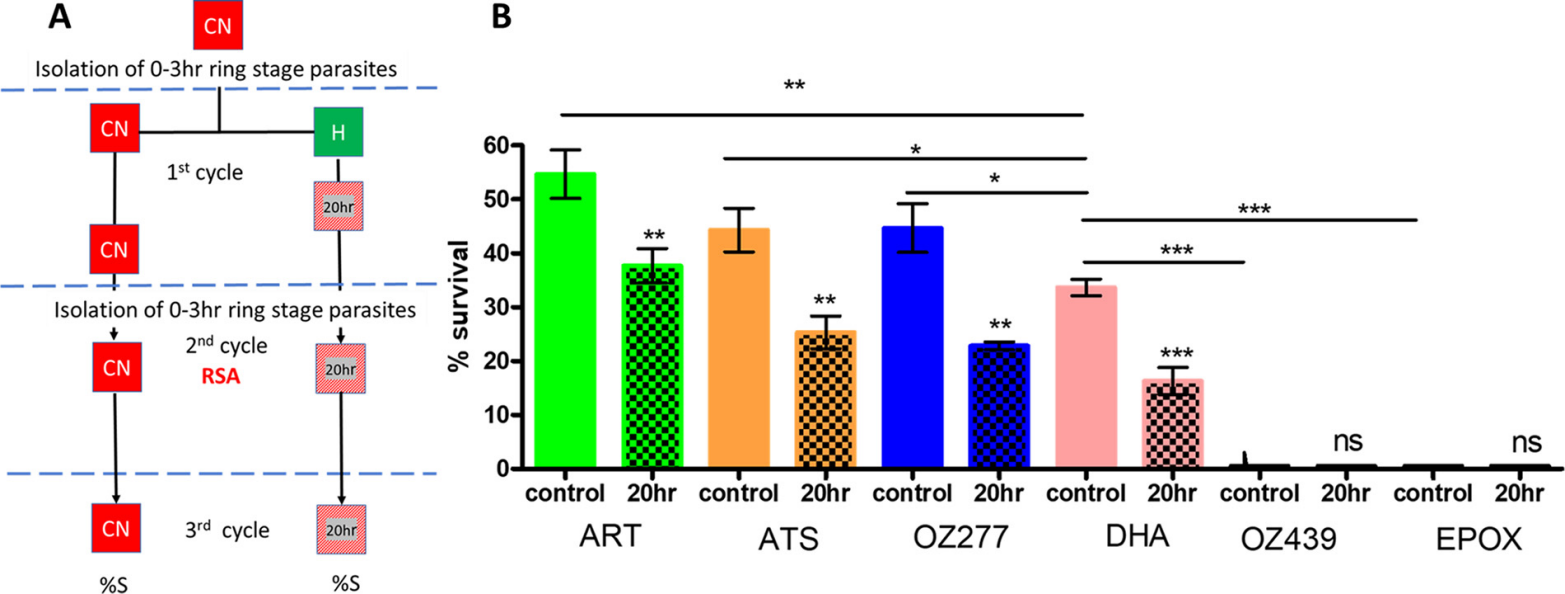
Effect of hyperoxic exposure on parasite tolerance to ART, ATS, OZ277, OZ439, and the proteasome inhibitor epoxomicin. (A) Experimental flow diagram. Continuous normoxia control (CN) 0- to 3-h ring-stage parasites were isolated and maintained in normoxia or exposed to hyperoxia for the first 20 h of ring stage development before returning to normoxia. In the second cycle of development, 0- to 3-h ring-stage parasites were used to evaluate the activity of DHA and other drugs in the RSA for both continuous normoxia (CN) and 20-h-hyperoxia-exposed ring-stage parasites. (B) Comparison of percent survival of Cam5 I543T to endoperoxides for continuous normoxia or 20 h hyperoxia. *P* values were calculated using one-tailed nonpaired Student’s *t* test (one directional) with 95% confidence limits using GraphPad Prism for reduction in %S in response to hyperoxia for each compound set. *, *P* < 0.05; **, *P* < 0.005; ***, *P* < 0.0005; ns, not significant. Values are means (*n* = 3) and SD.

To determine if ring-stage hyperoxia exposure of a group A strain (Cam5 I543T) elicited reduced parasite tolerance to endoperoxides in general, ART, ATS, and synthetic ozonides (OZ277 and OZ439) were assessed at 700 nM, the single dose used for DHA throughout this study. Epoxomicin (EPOX), a selective proteasome inhibitor previously observed to be equally active against K13 mutant and wild-type ring-stage parasites (38), was also evaluated at a dose of 70 nM (100× IC_50_) (0.74 nM) determined in a 72-h 3D7 imaging assay (61) as the dose for maximal killing of 0- to 3-h ring-stage parasites. The compounds were assessed for 0- to 3-h parasite (Cam5 I543T) survival after treatment with and without a 20-h pre-RSA hyperoxic exposure (Fig. 6A and B). Previous studies by Yang et al. (62) have demonstrated the requirement of more vigorous washing to negate potential compound carryover when testing ozonides in RSA-type evaluations. Therefore, the volume of medium used for compound removal after 5 h incubation was doubled from 50 mL to 100 mL for all compounds evaluated.

While ART, ATS, and OZ277 elicited responses comparable to that to DHA under hyperoxia, EPOX and OZ439 demonstrated complete killing of ring-stage parasites under both conditions tested. However, it must be noted that this study did not incorporate adjustments for clinically relevant dose and incubation times appropriate for each drug evaluated (63), which were not considered within the context of this study. These data, however, demonstrated that the effect was not limited to DHA. Hyperoxia effects on OZ439 and EPOX were not observable within this single high-drug-dose evaluation, as parasite survival under conditions of both normoxia and hyperoxia was less than 0.01%.

## DISCUSSION

Cam2 C580Y, Cam3.1 R539T, and Cam5 I543T (plus their reverted WT counterparts), i.e., group A, all contain 5 genetic background mutations (fd, crt, mdr2, arps10, and nif4) previously associated with predisposition to K13 mutation acquisition (12, 32). This study demonstrated that these mutations, as part of a wider, yet-to-be-identified genetic background (24, 25, 32), align with a hyperoxic stress tolerance phenotype (group A) that in turn modulates mutant K13 strain sensitivity to DHA, ART, ATS, and the synthetic ozonide OZ277. Due to the single-dose nature of the testing of compound activities within this study, the effect of hyperoxia on OZ439 and EPOX could not be determined, as both normoxic and hyperoxic conditions elicited the complete killing of Cam5 I543T. Extended investigations into OZ439 and EPOX in dose response and exposure times may uncover any effects of hyperoxia on the activities of these specific compounds.

This study utilized parasite strains obtained from BEI Resources (https://www.beiresources.org/), Cam2 C580Y, Cam3.1 R539T, and Cam5 I543T, which were not subcloned prior to evaluation. Both Cam2 C580Y and Cam3.1 R539T have subsequently been identified as multiclonal, consisting of at least 2 and 3 haplotypes, respectively (64). Using allele sharing simulations (65), Nkhoma and colleagues demonstrated that haplotypes from single clinical isolates were related, but those from different clinical isolate were not. From the study by Nkhoma et al. (64), drug sensitivity to chloroquine, mefloquine, and piperaquine determined in a 72-h standard sensitivity assay demonstrated sensitive, moderately resistant, and fully resistant profiles for the individual haplotypes from a single isolate. Of particular interest with respect to the data presented in this study (64), the determination of IC_50_s for chloroquine, mefloquine, and piperaquine in the standard 72-h assay was performed in hyperoxia, in contrast to *in vitro* culturing and RSA experiments, which were performed in normoxia (64). Thus, the impact of hyperoxia versus normoxia on the IC_50_s obtained between the haplotypes may also be an underlying effect on the variable haplotype drug sensitivities observed, as previously identified by Briolant et al. for clinical isolates (52).

The study by Nkhoma et al. also demonstrated that the three haplotypes obtained from Cam3.1 R539T resulted in DHA survival rates between 10 and 20%, while the rate for the parental mixed-clone isolate was approximately 50% (64). Within this study, survival after hyperoxic exposure for Cam3.1 R539T was 16.85% ± 6.5% and normoxia survival was 58.5% ± 11.2%, which could suggest a role of individual haplotypes in responding to hyperoxia. Unfortunately, no haplotype with parental strain DHA survival levels was isolated by Nkhoma and colleagues for Cam3.1 R539T (64). The isolation of a specific haplotype with survival levels comparable to that of the parent after DHA exposure in normoxia, with increased DHA sensitivity after hyperoxic exposure, would undoubtedly provide the tools necessary for investigating the mechanisms involved in parasite modulation of the K13-associated ability to survive DHA exposure, if indeed the response to hyperoxia is haplotype specific.

Based on the study by Nkhoma et al. (64), the data obtained herein may be the result of individual haplotypes (Cam3.1 R539T) modulating the DHA tolerance in normoxic conditions, working as a community for survival, with K13 mutations shared by all haplotypes from the same isolate defining the DHA tolerance under hyperoxic conditions (64). Alternatively, the observation of hyperoxia sensitization of parasites with naturally acquired K13 mutations to DHA, ART, ATS, and OZ277, the cycle prior to compound treatment, may have links to both parasite transcriptional control and the apicoplast.

The ability to withstand hyperoxic exposure and a loss of parasite tolerance to DHA was not an acute response but required 20 h exposure after RBC invasion. This suggested a controlled response by the parasite to counteract elevated stress in the first cycle, which was sufficient to maintain parasite proliferation after the second cycle. However, a full protective response to DHA exposure in the second cycle was prevented, resulting in only baseline survival of parasites (Fig. 5). Transcriptional variation in *Pf* is well documented (66–69), especially in relation to immune evasion, but also includes gene families exhibiting clonally variant expression of the response to environmental stress stimuli (69). Included are genes involved in protein folding, erythrocyte remodeling, lipid metabolism, or transcriptional regulation. Rovira-Graells et al. (69) proposed that *Pf* employs a “bet-hedging strategy” to counteract changes in environmental stimuli, where parasites with the “best set” of genes switched on or off at the time of the stimulus (i.e., the fittest) survive to the next cycle and those with other gene sets die. This bet-hedging strategy by *Pf* could be at play within the period of hyperoxic exposure among the strains investigated in this study. In WT parasites, the bet-hedging strategy would appear to fit with this scenario, whereas the parasites with delayed-PCT genetic background have adapted beyond this mechanism, as all parasites survive the stress stimulus with respect to proliferation. However, parasite survival of DHA challenge is reduced by hyperoxic exposure in the second proliferation cycle, but only minimally in the first.

This second-cycle consequence of stress is very reminiscent of AP2-G gametocytogenesis triggered by stress conditions. A smaller noncanonical pathway to gametocytogenesis occurs within the first cycle (70), with the major commitment occurring in the subsequent proliferation cycle, in alignment with the minor first-cycle and major second-cycle events witnessed within this study in response to hyperoxia. AP2-G is a member of the APiAP2 transcription factors/DNA binding proteins, which have been implicated in ART tolerance (71) and response to heat shock (72). Mok et al. (71) observed elevated expression levels of AP2-G2 (PF14_0079/PF3D7_1408200) in ART-resistant versus ART-sensitive strains. The association of APiAP2 transcription factors, ART resistance, and second-cycle effects on parasite development could suggest a potential role for altered transcriptional regulation occurring in the first 20 h of development in response to hyperoxic stress, which may underlie the reduced DHA tolerance in the next cycle.

This proposed hypothesis of a transcriptional response by group A strains, overriding standard responses to oxidative stress, is further strengthened by a second study by Zhu et al. (25) applying transcriptome wide association studies of patient samples pre- and posttreatment. The study identified a diverse group of biological functions, including redox metabolism, which was differentially altered in ART-resistant and -sensitive parasite isolates.

Regarding the genetic background associated with K13 mutations and ART tolerance, the breadth of research in this area is somewhat limited regarding functional analysis. However, the role of the apicoplast in ART tolerance is generating significant interest. Genetic mutations associated with ART tolerance include fd (32, 73–75) and arps10 (32), which are localized to the apicoplast, and ATG18, which, although it is not localized to the apicoplast, is associated with apicoplast biogenesis (76), suggestive of an integral role of the apicoplast in ART tolerance. In comparison, fd mutations (D193Y) inserted and removed from K13 mutant and WT strains by Stokes and colleagues (77) using gene insertion techniques exhibited no apparent effect on parasite survival. However, the study by Stokes and colleagues (77) employed normoxia culturing conditions, in which any effect contributed by ferredoxin mutations would not be observed, if fd involvement is only in response to a previous episode of hyperoxic stress, as proposed by this study.

A further strong link between fever, heat shock-responsive elements, the apicoplast, and ART tolerance has been identified (78). This suggests that *Pf* may adapt its innate fever response to resist ART-type drugs. It is therefore possible that the responses to fever, including those pertaining to the apicoplast, share a pathway utilized by other stress responses. It is thus anticipated that both the modulation of parasite proliferation and the reduction in parasite tolerance to DHA, ART, ATS, and OZ277, in response to hyperoxic stress, may share similar mechanisms while simultaneously involving individual distinct components.

Continuing investigations into the mechanisms underlying the modulation of DHA tolerance mediated via K13 mutations and associated genetic background are to include (i) single-cell cloning of Cam3.1 R539T and the identification of a DHA-tolerant hyperoxia-responsive haplotype, (ii) transcriptomics of hyperoxia-exposed K13 mutant and WT strains, and (iii) exploration of the relationship between the apicoplast, ferredoxin, and ART tolerance.

## MATERIALS AND METHODS

The *Plasmodium* strains (Table 1) were obtained from BEI Resources (Manassas, VA). The routine control culturing conditions used are published in detail elsewhere (63). Briefly, parasite cultures were maintained in RPMI 1640 medium supplemented with 10 mM HEPES, 50 μg/mL hypoxanthine, 2.5 mg/mL AlbuMAX II, and 5% human serum. Cultures were maintained between 1 and 7% parasitemia (P) (1 to 2% trophozoites maximum and 5 to 7% rings at various stages of the process) at 5% hematocrit (H) in petri dishes incubated at physiological normoxia (5% O_2_, 5% CO_2_, 37°C) in Tri-Gas incubators.

To evaluate the effect of hyperoxia on both parasite proliferation and tolerance to DHA and other drugs, cultures were maintained in constant normoxia (CN) for 7 to 12 days before starting each evaluation with ring-stage parasites. Highly synchronous 0- to 3-h ring stages were obtained by schizont isolation using magnetic column technology (63) and the addition of fresh RBC to isolated mature schizonts. After 3 h incubation, the culture was treated with 5% sorbitol to lyse unruptured schizonts, resulting in 0- to 3-h ring-stage parasite cultures. For experiments not requiring 0- to 3-h ring-stage parasites, sorbitol lysis was employed. To perform an evaluation, ring-stage parasites were isolated from the continuous normoxic control (CN), adjusted to 1% P at 5% H, and exposed to hyperoxia or normoxia during parasite development to schizont. At the schizont stage, ring-stage parasites were once more isolated and utilized in the proliferation and DHA tolerance studies. The evaluations in the second development cycle were performed at 1% P with H reduced to 2%, to align with that used in the RSA (7, 8). DHA at 700 nM for 5 h was used for evaluating the DHA tolerance utilizing the RSA. The P for the studies was determined by manual microscopy of Giemsa-stained blood smears after 65 h incubation (proliferation) or a total of 80 h for the RSA, i.e., within the third development cycle from initiation of the investigation. The percent *P* values obtained were then used to calculate the percent reduction in parasite proliferation in response to hyperoxia (Fig. 1 and 4), the percent reduction in parasite survival after DHA exposure in comparison to the normoxic control (CN) (Fig. 2 and 3), and the percent survival (%S) after DHA exposure (Fig. 5 and 6).

All experiments were performed using parasites from frozen stocks stored in liquid nitrogen, with all *in vitro* evaluations performed within 7 to 12 days of continuous culture to minimize *in vitro* culturing adaptations. A minimum of three independent biological replicates were performed for all analyses.
